# Geographic Clustering of Fast-Food Restaurants Around Secondary Schools in Hong Kong

**DOI:** 10.5888/pcd18.200601

**Published:** 2021-06-03

**Authors:** Jason Tsz Him Cheung, Ka Chung Tang, Keumseok Koh

**Affiliations:** 1 Division of Social Science, The Hong Kong University of Science and Technology, Kowloon, Hong Kong SAR, China; 2 Department of Geography, The University of Hong Kong, Pokfulam, Hong Kong SAR, China

## Abstract

**Introduction:**

Clustering of fast-food restaurants around schools facilitates fast-food consumption among students, which may cause obesity. We examined the prevalence of fast-food restaurants and identified the clusters of fast-food restaurants near secondary schools in Hong Kong.

**Methods:**

We collected data of Western fast-food chain restaurants and 490 secondary schools in Hong Kong. Descriptive statistics and buffer analysis identified the prevalence of fast-food restaurants around the secondary schools within 400-m and 800-m buffers. Additional analyses compared schools stratified by the 3 main regions in Hong Kong, district-level population density, and median monthly household income. We used Getis-Ord GI* hot spot analysis to measure spatial clusters of fast-food restaurants around schools and Global Moran’s *I* to measure the spatial autocorrelation based on each school and the number of fast-food restaurants within the 400-m buffer.

**Results:**

The average number of fast-food restaurants within 400 m and 800 m of a school was 2.0 and 6.3, respectively. Seven in 10 secondary schools had at least 1 fast-food restaurant within 400 m. The number of schools with no fast-food restaurants was higher in Hong Kong Island, considered the “rich region” in Hong Kong. Hot spots of clusters were significantly located in the high-density downtown areas. We observed significant spatial autocorrelation between fast-food restaurants and secondary schools in the areas with high density, low income, and high income (*P* < .001, z > 2.58).

**Conclusion:**

Fast-food restaurants were substantially clustered around secondary schools in Hong Kong. Territory-wide studies about the health effect of fast-food clusters around schools on children and adolescents are warranted in Hong Kong.

**Summary**

**What is already known on this topic?**

Previous literature confirmed a positive association between clusters of fast-food restaurants around schools and obesity rates of school children. Few studies about fast-food clustering around schools have been conducted in a high-density urban setting such as Hong Kong.

**What is added by this report?**

Fast-food restaurants around secondary schools were substantially clustered in Hong Kong. The school food environment in Hong Kong showed unique heterogeneities compared with the school food environment in a Western setting.

**What are the implications for public health practice?**

Students in some areas may have a disproportionate share of health problems caused by an obesogenic environment. Observed clusters of fast-food restaurants in Hong Kong can alert policy makers to design effective interventions targeting the secondary schools located in such environments.

## Introduction

Childhood obesity is a major public health problem in both developed and developing countries (1). In 2015, a total of 107.7 million children were obese across the world, and the rate of increase among children exceeded that among adults (2). The long-term physiological and psychological consequences of obesity include an increased risk of type 2 diabetes, high blood pressure, high cholesterol, fatty liver disease, adverse cardiovascular outcomes, low self-esteem, anxiety, depression, bullying, and stigma, which are likely to persist into adulthood (3–6).

The World Health Organization warned that excessive consumption of a high-fat diet is associated with childhood obesity (7). Fast food is one of the dominant food types constituting a high-fat diet. The consequences of fast-food consumption on children’s dietary intake are serious. Compared with nonconsumers of fast food, children who consume fast food were found to have higher intakes of total energy, total fat, saturated fat, and sugar and lower intakes of fiber, fruit, vegetables, and milk (8). In the long run, frequent fast-food consumption among children was associated with a higher body mass index (BMI), higher body fat percentage, and increased odds of being obese (9).

The school food environment is an important factor in shaping children’s food choices, along with other factors, such as levels of physical activity, caregivers’ income, and social and cultural knowledge and attitudes (10). The rapid expansion of Western fast-food restaurants in urban environments in high-income countries and their increased availability, accessibility, and affordability are associated with the emergence of childhood obesity (11). One study found that children with more fast-food restaurants in their school neighborhoods ate more fast-food meals per week (12). Public health and nutrition professionals recommend various school-based dietary interventions (eg, school lunch programs and nutrition education) to mitigate childhood obesity; such interventions can reduce BMI and the prevalence of childhood obesity (13).

School-based dietary intervention programs are not always effective. In Hong Kong, although most primary schools have dietary intervention programs, secondary school students (particularly seniors) often enjoy having lunch at nearby restaurants. The food choices of secondary school students are often driven by accessibility, availability, affordability, acceptability (eg, perception of their school’s food), and attitudes concerning food (14). In Hong Kong, Western fast-food is perceived as “cool” by young people, who also praise its taste, appeal, affordability, and quickness (14–16). In addition, Hong Kong’s hyper-dense urban nature gives students an additional benefit — proximity — to fast-food restaurants. The proximity of fast-food restaurants to secondary schools is a crucial factor in determining the daily food choices of students. The overall overweight and obesity rate among secondary school students in Hong Kong increased from 18.2% in school year 2009–2010 to 19.9% in 2018–2019 (17). Nearly 1 in 5 secondary students is overweight or has obesity (17).

To the best of our knowledge, only a few studies have examined the prevalence of fast-food restaurants near secondary schools in Hong Kong (18). Also, few government policies have solely addressed the problem of fast-food restaurants near secondary schools and its health effect on children (18). Therefore, this study aimed to describe the prevalence of fast-food restaurants near secondary schools and identify the vulnerable hot spots of fast-food restaurant clusters near secondary schools.

## Methods

We performed a cross-sectional analysis in July 2020 using up-to-date, publicly available data on fast-food restaurants and secondary schools in Hong Kong.

**Figure 1:**
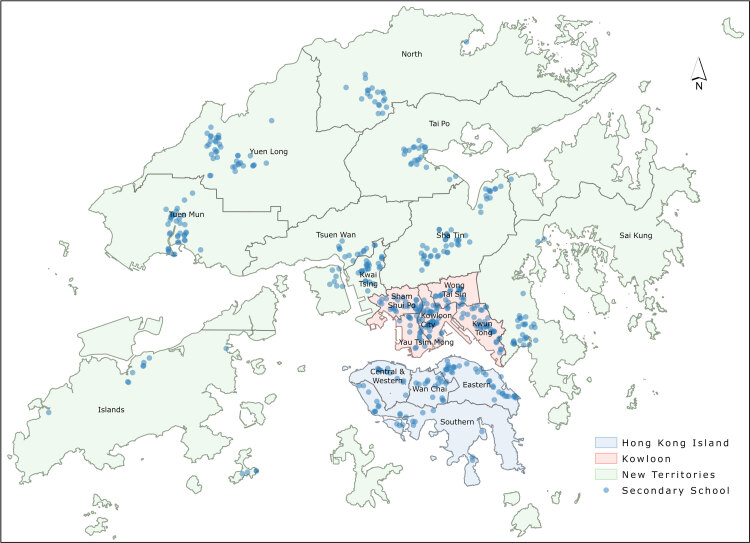
The location of 490 secondary schools in 3 regions and 18 districts in Hong Kong.

**Hong Kong base map data.** Hong Kong comprises 3 regions and 18 districts, namely, Hong Kong Island (4 districts), Kowloon (5 districts), and New Territories (9 districts) (Figure 1). We used district-level data to examine the overall prevalence of fast-food restaurants around each secondary school.

**Fast-food restaurant data.** We compiled the latest list of fast-food restaurants in Hong Kong in July 2020 from a popular and comprehensive website for food and restaurant reviews in Hong Kong, OpenRice (www.openrice.com). We searched the OpenRice fast-food category for the names of major, if not all, Western fast-food chain restaurants (ie, restaurants that quickly serve food such as hamburgers, French fries, fried chicken, submarine sandwiches, and pizza). The 15 fast-food chain restaurants included in the analysis were Burger Circus, Burger Home, Burger King, Burgerman, BurgerRoom, Five Guys, Jollibee, KFC, McDonald’s, Moo-Moo, Mos Burger, Popeyes, Subway, Texas Burger, and The Big Bite. We excluded Hong Kong–style fast-food restaurants (eg, Café de Coral, Maxim’s MX) because they offer many different styles of cuisines (18). We collected up-to-date street addresses from the official website of each fast-food restaurant. We ensured that the restaurants were in business during the time of our analysis (July 2020) and geocoded the street addresses using Google Maps Geocoding Application Programming Interface (API). All records were successfully geocoded. After geocoding, we conducted reverse geocoding to ensure addresses were accurate.

**EatSmart restaurant data. **In Hong Kong, to advocate for a healthy food environment, the government initiated the EatSmart Restaurant Star campaign in 2008 (19). The campaign uses a label system to indicate the level of fruits and vegetables served to customers. Only 972 of 16,323 restaurants in Hong Kong, and none of the fast-food restaurants analyzed in our study, joined this campaign as of December 2020. To allow the comparison of the spatial variations of fast-food restaurants and the EatSmart restaurants around secondary schools, we collected the addresses of the EatSmart restaurants from the EatSmart Restaurant Star campaign official website in December 2020 (19). We geocoded the street addresses using Google Maps Geocoding API. All records were successfully geocoded. After geocoding, we conducted reverse geocoding to ensure addresses were accurate.

**Secondary school data.** We obtained secondary school data in 2020 from the Hong Kong GeoData Store, a free geospatial information service run by the Hong Kong government, by using the location search API (20). We included all secondary schools in Hong Kong: aided secondary schools, Caput secondary schools, direct subsidy scheme secondary schools, English Schools Foundation (secondary), government secondary schools, international schools (secondary), and private secondary schools (day/evening). After retrieving the addresses, we compiled a database with 490 listings geocoded by Google Maps Geocoding API. We conducted reverse geocoding to ensure addresses were accurate.

**Census data.** We collected data on population density and median monthly household income at the district level from the most recent (2016) Hong Kong government census (21,22).

### Statistical analyses

First, we calculated the mean and median distance from each secondary school to the closest fast-food restaurant. Second, we conducted 400-m and 800-m Euclidean buffer analyses around each school and identified the number of fast-food restaurants within the buffers. The 400-m Euclidean buffer reflects the reality of students walking to nearby restaurants (23). The 800-m Euclidean buffer was used to characterize a larger school food environment, targeting students who exert extra effort and intention to visit fast-food restaurants (24). Third, we conducted the Getis-Ord GI* hot spot analysis to identify the significant spatial clusters of fast-food restaurants. We adopted the inverse distance-weighted interpolation to visualize the hot spots according to *z* scores, which indicate the significance of the hot spot. A low negative *z* score generally implies a cold spot, a high positive *z* score implies a hot spot, and a *z* score near zero indicates no apparent spatial clustering. Finally, to quantify the degree of clustering, we used the spatial autocorrelation (Global Moran’s *I*) tool to measure the spatial autocorrelation based on each school and the number of fast-food restaurants within the 400-m buffer. To allow for the comparison of the geographic clustering of fast-food restaurants and EatSmart restaurants around secondary schools, we also performed the same spatial autocorrelation (Global Moran’s *I*) analysis based on each school and the number of EatSmart restaurants within the 400-m buffer. This tool evaluates whether the pattern is clustered, dispersed, or random, where a Moran’s *I* value near +1 indicates clustering and a Moran’s *I* value near −1 indicates dispersion. The Moran’s *I* statistic for spatial autocorrelation is given as

$I=\;\frac{N}{S_{0}}\sum_{i}\sum_{j}w_{ij}\frac{\left(x_{i}-\;\mu \right)\left(x_{j}-\;\mu \right)}{\sum_{i}({x_{i}-\;\mu )}^{2}}$

where N is the number of schools; *w*_ij_ is the element in the spatial-weight matrix corresponding to the samples *i* and *j*; *w*_ij_ is defined using an inverse distance method; and *x*_i_ and *x*_j_ are samples for areas *i* and *j* with the mean μ; and

$S_{0}=\;\sum_{i}\sum_{j}w_{ij}$

All analyses were carried out by using ArcGIS Pro version 2.4.0 (Esri).

We analyzed the data from Hong Kong as a whole and then conducted 3 additional stratified analyses. First, we examined the spatial characteristics of the 3 regions. Second, we classified the 18 districts into 3 strata of population density (high, medium, and low) and analyzed the spatial characteristics of the 3 strata. The 3 strata were classified according to the relative rankings of population density in the 18 districts. We then assigned each school to 1 of the 3 strata of population density, according to their district. Third, we classified the 18 districts into 3 strata of median monthly household income (high, medium, and low) and analyzed the spatial characteristics of the 3 strata. The 3 strata were classified based on the relative rankings of median monthly household income in the 18 districts. We then assigned each school to 1 of the 3 strata of median monthly household income, according to their district.

## Results

We found 425 Western fast-food chain restaurants in Hong Kong: Burger Circus (n = 1; 0.2%), Burger Home (n = 1; 0.2%), Burger King (n = 2; 0.5%), Burgerman (n = 2; 0.5%), BurgerRoom (n = 2; 0.5%), Five Guys (n = 4; 0.9%), Jollibee (n = 10; 2.4%), KFC (n = 87; 20.5%), McDonald’s (n = 247; 58.1%), Moo Moo (n = 1; 0.2%), Mos Burger (n = 33; 7.8%), Popeyes (n = 1; 0.2%), Subway (n = 31; 7.3%), Texas Burger (n = 1; 0.2%), and The Big Bite (n = 2; 0.5%).

Overall, in Hong Kong, the mean and median distance between each school and the most proximate fast-food restaurant were 377.0 m and 278.7 m, both within the 400-m walkable distance (Table 1). The average number of fast-food restaurants within 400 m and 800 m of a school was 2.0 and 6.3, respectively (Table 2). Approximately 7 in 10 secondary schools (72.0%) had at least 1 fast-food restaurant within 400 m, and half (52.0%) had more than one. Across the 3 regions, Kowloon had the highest average number of fast-food restaurants within 400 m and 800 m of a secondary school. More than 7 in 10 secondary schools had at least 1 fast-food restaurant within 400 m in Kowloon (76.4%) and New Territories (72.0%). In contrast, Hong Kong Island, which has the highest median monthly household income across the 3 regions, had fewer fast-food restaurants near secondary schools, yet 64.8% had at least 1 fast-food restaurant and 51.1% had more than 1 fast-food restaurant within 400 m. The number of schools with no fast-food restaurants within 400 m was higher in Hong Kong Island (35.2%) than in Kowloon (23.6%) or New Territories (28.0%).

**Table 1:** Number of Secondary Schools and Fast-Food Restaurants and Distance Between Each School and Nearest Fast-Food Restaurant, by Region and District Characteristics, Hong Kong, 2020

Region and Characteristic	No. of Schools	No. of Fast-Food Restaurants	Distance, m			
			Mean	Median	SD	Range
**Hong Kong overall**	490	531	377.0	278.7	484.4	32.4–7955.2
**By region**						
Hong Kong Island	88	119	416.0	274.8	402.8	32.4–2502.6
Kowloon	148	183	302.0	275.3	165.9	57.1–848.0
New Territories	254	229	409.2	279.0	614.6	56.3–7955.2
**By population density^a^**						
Low	137	126	502.7	270.1	869.7	56.3–7955.2
Middle	171	183	383.9	287.2	323.2	32.4–2502.6
High	182	222	307.3	277.4	171.0	40.2–859.3
**By household income^b^**						
Low	177	172	387.0	305.7	213.7	57.1–1517.3
Middle	183	187	384.1	279.2	429.5	56.3–3749.3
High	130	172	380.4	245.8	370.2	32.4–2502.6

a The 3 strata of population density were classified based on the relative rankings of population density in the 18 districts of Hong Kong.

b The 3 strata of household income were classified based on the relative rankings of median monthly household income in the 18 districts of Hong Kong.

**Table 2:** Number of Fast-Food Restaurants Within 400-m and 800-m Buffer of Each Secondary School (N = 490), by Region and District Characteristics, Hong Kong, 2020

Region and Characteristic	400-m Buffer				800-m Buffer			
	Mean No. of Restaurants	Schools With 0 Restaurants, No. (%)	Schools With at Least 1 Restaurant, No. (%)	Schools With >1 Restaurant, No. (%)	Mean No. of Restaurants	Schools With 0 Restaurants, No. (%)	Schools With at Least 1 Restaurant, No. (%)	Schools With >1 Restaurant, No. (%)
**Hong Kong overall**	2.0	137 (28.0)	353 (72.0)	255 (52.0)	6.3	29 (5.9)	461 (94.1)	435 (88.8)
**By region**								
Hong Kong Island	2.0	31 (35.2)	57 (64.8)	45 (51.1)	6.2	9 (10.2)	79 (89.8)	76 (86.4)
Kowloon	2.2	35 (23.6)	113 (76.4)	80 (54.1)	8.5	1 (0.7)	147 (99.3)	141 (95.3)
New Territories	2.0	71 (28.0)	183 (72.0)	130 (51.2)	5.2	19 (7.5)	235 (92.5)	218 (85.8)
**By population density^a^**								
Low	2.0	41 (29.9)	96 (70.1)	70 (51.1)	5.1	17 (12.4)	120 (87.6)	111 (81.0)
Middle	1.9	51 (29.8)	120 (70.2)	85 (49.7)	5.5	11 (6.4)	160 (93.6)	149 (87.1)
High	2.2	45 (24.7)	137 (75.3)	100 (54.9)	8.1	1 (0.5)	181 (99.5)	175 (96.2)
**By household income^b^**								
Low	1.9	48 (27.1)	129 (72.9)	86 (48.6)	6.4	6 (3.4)	171 (96.6)	163 (92.1)
Middle	2.0	49 (26.8)	134 (73.2)	98 (53.6)	6.4	11 (6.0)	172 (94.0)	159 (86.9)
High	2.3	40 (30.8)	90 (69.2)	71 (54.6)	6.2	12 (9.2)	118 (90.8)	113 (86.9)

a The 3 strata of population density were classified based on the relative rankings of population density in the 18 districts of Hong Kong.

b The 3 strata of household income were classified based on the relative rankings of median monthly household income in the 18 districts of Hong Kong.

For secondary schools within the districts stratified by population density, secondary schools in high-density districts had a higher percentage of having at least 1 fast-food restaurant and more than 1 fast-food restaurant compared with secondary schools in middle-density and low-density districts (table 2). Three-quarters of secondary schools (75.3%) in high-density districts had at least 1 fast-food restaurant within 400 m. For secondary schools stratified by median monthly household income, secondary schools in low-income (72.9%) and middle-income (73.2%) districts generally had a higher percentage of having at least 1 fast-food restaurant within 400 m. Secondary schools in high-income districts had a higher percentage of having more than 1 fast-food restaurant within 400 m (54.6%).

**Figure 2:**
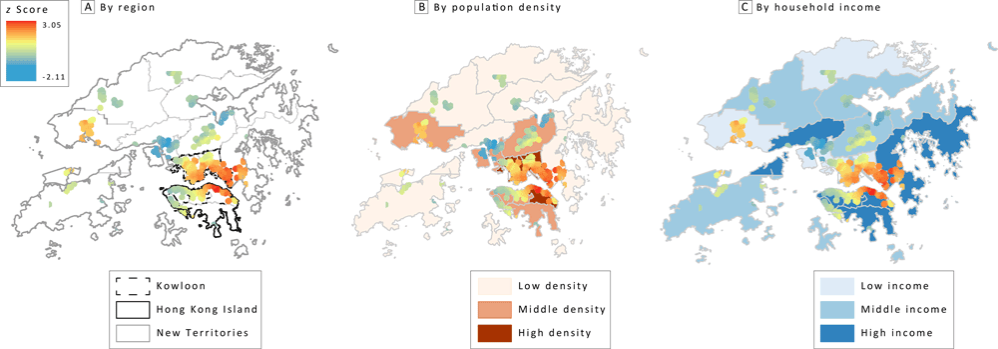
Hot spot analysis of fast-food restaurants near secondary schools in Hong Kong, by A, region, B, population density, and C, income.

In the hot spot analysis, we observed that significant clusters were in the high-density downtown areas of Hong Kong (Figure 2). Most hot spots were in Kowloon. The secondary schools in Eastern and Wan Chai districts in Hong Kong Island also were exposed to a significantly high number of clusters of fast-food restaurants. In New Territories, we found fewer clusters of fast-food restaurants around the secondary schools; Tuen Mun district was an exception. In the spatial autocorrelation analysis, we found that clusters of fast-food restaurants around each school were significant in most districts. In sum, fast-food restaurants and secondary schools in high-density urban areas in both low-income and high-income districts had significant spatial autocorrelations (*z* > 2.58; *P* < .001). The only exception for significant clustering of fast-food restaurants was the low-density districts (Table 3). In contrast, the degree of clustering of EatSmart restaurants was significantly lower in most regions and districts compared with that of fast-food restaurants. Although we observed significant clustering of EatSmart restaurants in high-density urban areas in both low-income and high-income districts (*z* > 2.58; *P* < .001), we observed no significant clustering of EatSmart restaurants in middle-income or middle-density districts (Table 3). This result suggests that students in middle-income and middle-density districts may be exposed to clusters of fast-food restaurants with no clusters of EatSmart restaurants to balance the food options.

**Table 3:** Spatial Autocorrelation (Global Moran’s Index) Based on Each School and the Number of Fast-Food Restaurants and EatSmart Restaurants^a^ Within a 400-m Buffer, By Region and District Characteristics, Hong Kong, 2020

Region and Characteristic	Fast-Food Restaurants			EatSmart Restaurants		
	Global Moran’s Index	*z* Score	*P* Value	Global Moran’s Index	*z* Score	*P* Value
**Hong Kong overall**	0.26	3.30	<.001	0.19	2.41	.02
**By region**						
Hong Kong Island	0.58	8.21	<.001	0.08	1.24	.21
Kowloon	0.32	6.24	<.001	0.39	7.73	<.001
New Territories	0.53	2.51	.01	0.32	1.58	.11
**By population density^b^**						
Low	0.57	1.66	.10	0.34	1.05	.30
Middle	0.67	2.29	.02	0.41	1.41	.16
High	0.28	6.87	<.001	0.34	8.32	<.001
**By household income^c^**						
Low	0.14	4.97	<.001	0.18	6.31	<.001
Middle	0.52	2.30	.02	0.37	1.68	.09
High	0.48	8.34	<.001	0.23	4.31	<.001

a Restaurants that adopted a labeling system indicating the level of fruits and vegetables served to customers.

b The 3 strata of population density were classified based on the relative rankings of population density in the 18 districts of Hong Kong.

c The 3 strata of household income were classified based on the relative rankings of median monthly household income in the 18 districts of Hong Kong.

## Discussion

To the best of our knowledge, our study is one of the first to examine the fast-food environment around secondary schools in Hong Kong. The main objective of this study was to describe the prevalence of fast-food restaurants near secondary schools and identify the hot spots of fast-food restaurants by using publicly available geospatial data. Our results suggest that fast-food restaurants around secondary schools were clustered in Hong Kong as a whole and almost all districts stratified by 3 main regions in Hong Kong, population density, and median monthly household income. The overall prevalence of overweight and obesity among secondary school students increased from school year 2009–2010 to school year 2018–2019 (17), consistent with the pattern observed in previous studies (25). Our study adds to the data on obesogenic school food environments in Hong Kong and complements the existing evidence by suggesting the uniqueness of the school food environment in highly compact cities in an East Asian context compared with Western settings, which are characterized generally by relatively low-density food environments (26).

Obesity rates are associated with fast-food restaurant clusters around schools; high school students with easy access to fast-food restaurants from schools have a higher probability of becoming obese (26). Our findings show that Hong Kong secondary school students are potentially exposed to substantial amounts of fast-food restaurants, particularly students who study in high-density urban areas in both low-income and high-income districts. On average, around each secondary school, we found 2.0 fast-food restaurants within 400 m and 6.3 fast-food restaurants within 800 m; students could easily walk to these fast-food restaurants. Policy makers may need to develop policies to improve the food environment near secondary schools, considering teenagers may be easily lured to consume unhealthy food in fast-food restaurants through peer influence (27). Most secondary school students, regardless of their financial resources, may find fast food more appealing because of its affordability, “coolness,” taste, and convenience (14,15). This appeal may explain why fast-food restaurants were strategically located near secondary schools in both low-income and high-income districts (28), although we also found a moderate cluster among secondary schools in the middle-income districts.

Furthermore, the number of fast-food restaurants around each secondary school in Hong Kong was higher than the number in Western settings (for example, the US) (26). This phenomenon could be attributable to the density of Hong Kong and its gentrification and redevelopment process in some of the older districts such as Kwun Tong and Kowloon City. We found many fast-food restaurant clusters in those redeveloped districts; thus, appraising accessibility to fast-food is crucial in redevelopment initiatives. The government may need to intensively formulate urban policies (eg, incorporating the concept of healthy eating into redevelopment plans) to mitigate potential health inequalities caused by geographic disparity. In Canada, urban planning policies such as zoning bylaws have been proliferated to ban fast-food restaurants (29). These full or partial bans emerged recently in North America to encourage healthier food options. Hong Kong has lacked zoning regulations or restrictions limiting the placement of fast-food restaurants (18). However, instead of a one-way tough-policy approach, the government could consider adopting the concept of zoning by providing incentives (eg, lower rents for places with more population flow) to reward fast-food restaurants that place their restaurants in a nonschool zone. In the long run, the Hong Kong government should initiate the discussion of zoning regulations for fast-food establishments around schools for long-term sociospatial sustainability, especially targeting the districts undergoing redevelopment.

In the autocorrelation analysis of EatSmart restaurants, our results (ie, no significant clustering of EatSmart restaurants in low-density and middle-density districts) may have been due to the strategic placement of restaurants in downtown areas with more population flow. To combat the pervasive clustering of fast-food restaurants, more EatSmart healthy restaurants located near secondary schools are warranted, especially in nondowntown areas. The EatSmart campaign should be further strengthened by fostering collaborations between the government and the food industry by increasing advertisement of this program and implementing attractive reward mechanisms for participating restaurants.

The government in Hong Kong has also attempted to address the problem of childhood obesity and obesogenic school food environments by launching EatSmart@school.hk, which consists of 3 main components: EatSmart School Accreditation Scheme, Salt Reduction Scheme for School Lunches, and Joyful Fruit Month (30). However, this program focuses only on primary school students. Secondary school students in Hong Kong have received limited support for nutrition intake interventions from the government, and Hong Kong’s public health community has expressed concerns about fast-food exposure among children and adolescents. Our study serves to raise awareness among authorities about food environments around secondary schools in Hong Kong. In the US, the National School Lunch Program was implemented in 1946 to enhance students’ nutritional intake by offering school meals which meet US Department of Agriculture standards: currently, these standards include increasing the number and variety of fruits, vegetables, and whole grains and reducing the intake of trans-fat content and calories (31). This school meal program reduced weight status among school lunch participants, especially among students eligible for subsidies (31). The Hong Kong government could consider formulating a school lunch program with high nutrition standards and subsidizing secondary school students to buy healthy school lunches. Apart from incorporating incentives to encourage students to eat nutritious food for lunch, the government could also initiate policies to lower the appeal of fast food by restricting fast-food advertising in major media channels and holding frequent educational sessions on the consequences of fast-food intake (32,33).

Our study has several limitations. We used Euclidean distance to assess distance between secondary schools and fast-food restaurants. In Hong Kong, which has many hills and short cuts, the distance used in the analysis may not reflect the real network distance between secondary schools and fast-food restaurants. However, given the generally high number of fast-food restaurants around secondary schools, the problem of Euclidean distance is not a major flaw in the interpretation of the severity of fast-food restaurant clusters in Hong Kong. Also, Euclidean buffers may allow a snapshot comparison of fast-food restaurant clustering around schools between Hong Kong and Western settings, because the use of network distance of Hong Kong may not provide a consistent basis for comparison because of the hilly, compact, and crowded nature of the streets. In addition, we only included the major Western fast-food chain restaurants and excluded the Hong Kong–style fast-food restaurants in our analysis; however, Hong Kong–style fast-food is also criticized because of its poor nutritional value.

The strengths of our study are twofold. It is one of the pioneering studies to examine the prevalence of fast-food restaurants near secondary schools in Hong Kong. Our findings can be used by the public health community and government officials to formulate strategic plans and interventions to improve the unhealthy school food environment in problematic areas. Also, our study provides a new approach to interpreting the school food environment in a compact urban setting, which potentially shows differences when compared with the Western setting, where the density of the food environment is generally lower (26). Our findings highlight the need for further developing theories and models for the school food environment in high-density cities or countries.

Secondary school students are constantly exposed to substantial amounts of fast-food restaurants in Hong Kong. The geographic clustering of fast-food restaurants around secondary schools should be addressed, and policy makers should pay attention to the consequences of this health problem as they unfold. In the near future, evidence from territory-wide cross-sectional and longitudinal studies about the health effects of fast-food restaurant clusters on school children are warranted.
